# Terpenoids from Marine Soft Coral of the Genus *Lemnalia*: Chemistry and Biological Activities

**DOI:** 10.3390/md16090320

**Published:** 2018-09-09

**Authors:** Qihao Wu, Jiadong Sun, Jianwei Chen, Huawei Zhang, Yue-Wei Guo, Hong Wang

**Affiliations:** 1 College of Pharmaceutical Sciences, Zhejiang University of Technology, Hangzhou 310014, China; qihaowu@zjut.edu.cn (Q.W.); cjw983617@zjut.edu.cn (J.C.); hwzhang@zjut.edu.cn (H.Z.); 2 State Key Laboratory of Drug Research, Shanghai Institute of Materia Medica, Chinese Academy of Sciences, 555 Zu Chong Zhi Road, Zhangjiang Hi-Tech Park, Shanghai 201203, China; 3 Laboratory of Bioorganic Chemistry, National Institute of Diabetes and Digestive and Kidney Diseases (NIDDK), National Institutes of Health, Bethesda, MD 20878, USA; sunjiadong@gmail.com

**Keywords:** marine soft coral, *Lemnalia* sp., secondary metabolites, bioactivity

## Abstract

*Lemnalia* is one of the most widely-distributed marine soft coral in tropical oceans and is known to produce novel terpenoids with a broad spectrum of biological activities. This review provides the first comprehensive overview of terpenoids produced by soft coral *Lemnalia* since their first discovery in 1974.

## 1. Introduction

Marine soft corals continue to be a rich source for the discovery of structurally diverse terpenoids with potential therapeutic applications [1,2,3]. In recent years, many marine soft corals distributed in the oceans from tropic to polar regions, such as Taiwan and South China Sea, have been chemically investigated and studied for potentially bioactive chemical components [4,5,6]. Among them, marine soft coral of the genus *Lemnalia* (Coelenterata, Octocorallia, Alcyonacea) has afforded numerous novel terpenoids with diverse chemical structures, including sesquiterpenes and diterpene glycosides with promising biological activities. Diverse biological properties enabled the *Lemnalia*-derived marine natural products (MNPs), mainly terpenoids, to attract attention of natural product chemists for the discovery of new drug candidates.

In order to exploit the MNPs of marine soft coral *Lemnalia* and better understand the medicinal significance of terpenoids from this genus, this review summarizes the chemical and/or biological investigations of soft coral *Lemnalia*. It covers topics ranging from the distribution of *Lemnalia* to the isolation, biosynthesis, and biological activities of different types of terpenoids, within a literature survey from 1974 to the present.

## 2. Marine Natural Products from *Lemnalia* sp.

**Figure 1:**
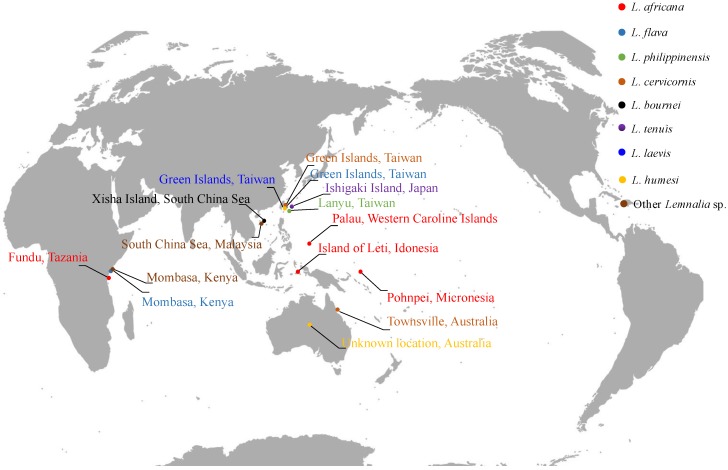
Global distribution of the chemically investigated *Lemnalia* marine soft coral according to their species.

**Scheme 1:**
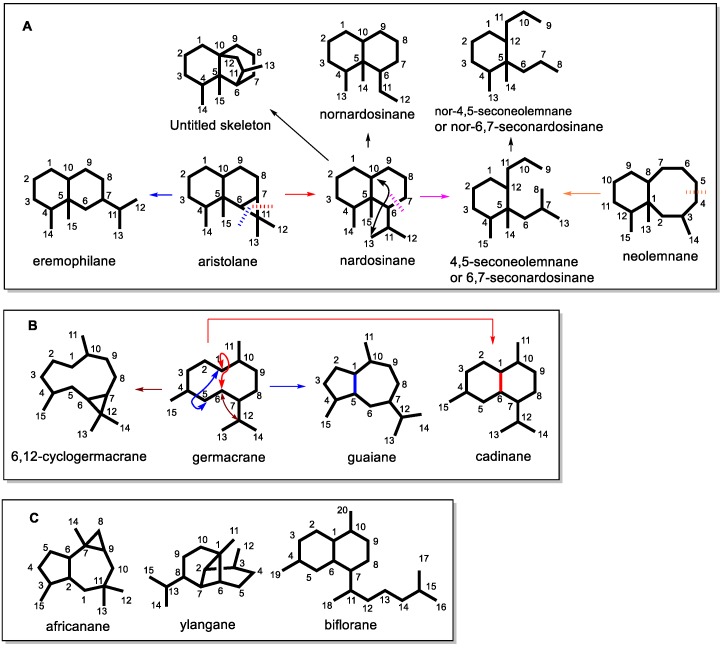
Various carbon skeletons of metabolites from soft coral of the genus *Lemnalia*. (**A**) Aristolane related carbon skeletons from soft coral *Lemnalia*; (**B**) Germacrane related carbon skeletons from soft coral *Lemnalia*; (**C**) Other carbon skeletons from soft coral *Lemnalia*.

**Figure 2:**
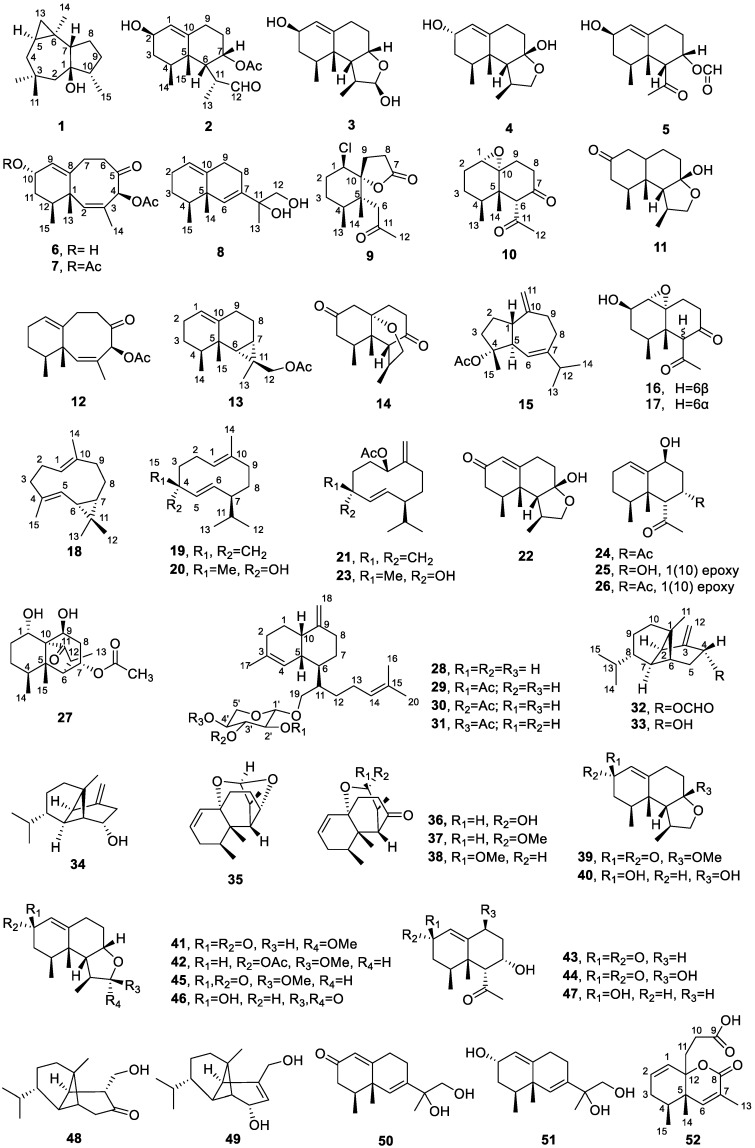
Chemical structures of compounds **1**–**52**.

**Figure 3:**
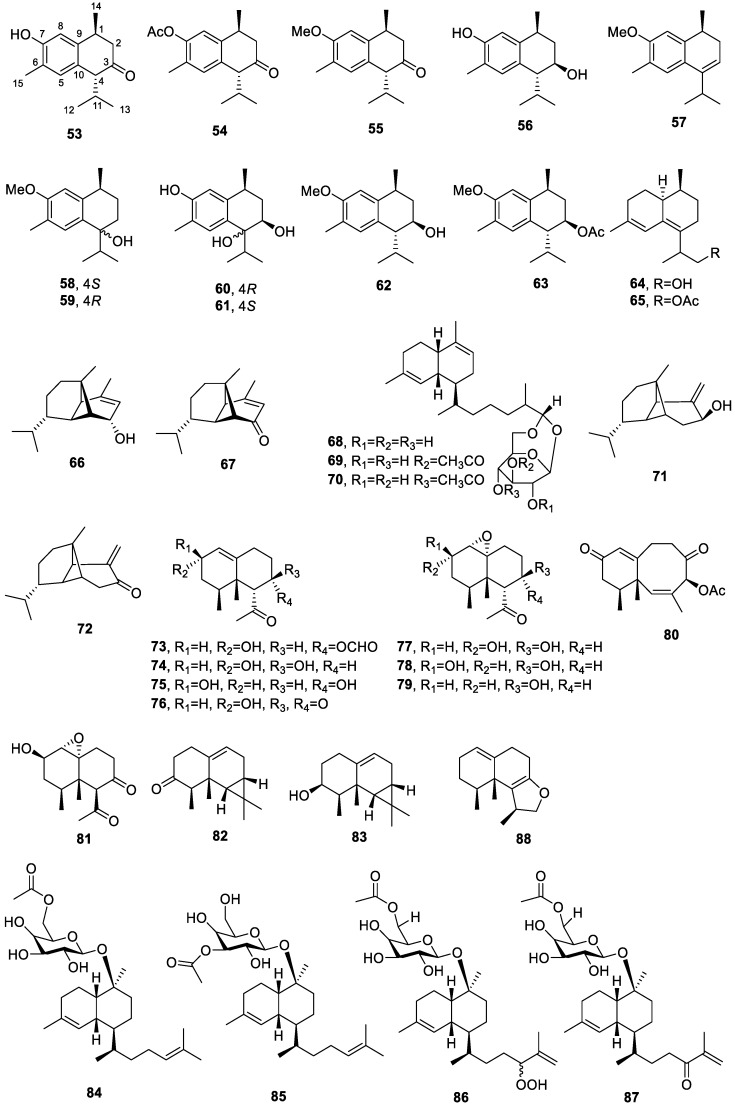
Chemical structures of compounds **53**–**88**.

**Figure 4:**
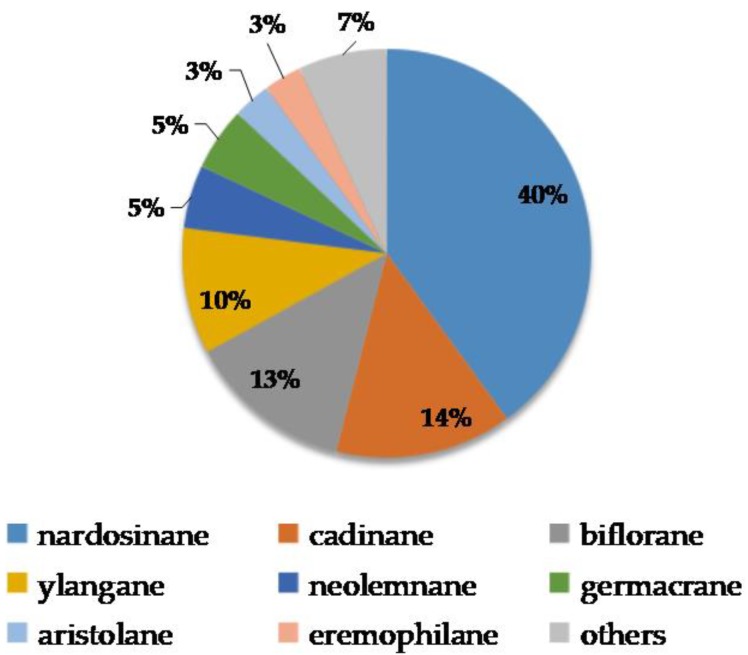
Terpenoids discovered from *Lemnalia* with different ratio of structural types.

Soft corals of the genus *Lemnalia* are distributed all over the oceans. Many of them were chemically investigated, especially those from the South China Sea, Taiwan, and off the coast of Australia and Kenya (Figure 1). Up to 2017, 88 terpenoids (Figure 2 and Figure 3) have been isolated and identified from the marine soft coral of the genus *Lemnalia*, including *L. africana*, *L. flava*, *L. philippinensis*, *L. cervicornis*, *L. bournei*, *L. tenuis*, *L. laevis*, *L. humesi*, *L. carnosa*, and other unidentified *Lemnalia* sp. (Table 1). Among all the terpenoids discovered from *Lemnalia*, 70 of them were found from *Lemnalia* for the first time, and the other 18 compounds were known from other sources. Sesquiterpenoids are the most prominent metabolites from *Lemnalia*, which can be classified into 14 types, such as nardosinane-type, neolemnane-type, ylangane-type, and other type sesquiterpenoids according to the carbon skeletons of their chemical structures (Scheme 1). Besides, diterpenoids from *Lemnalia* have a biflorane-based skeleton. As demonstrated in Figure 4, terpenoids were discovered and characterized by different ratio of structural types. Nardosinane-type sesquiterpenoids (40%) were commonly considered as chemotaxonomic markers of soft corals of genus *Lemnalia* (Table 1 and Figure 4); Cadinane-type sesquiterpenoids (14%) were mainly discovered from one soft coral sample collected off Broadhurst Reef, Townsville, Australia; Biflorane-type diterpenoids (13%) were mainly obtained from South China Sea; Ylangane-type sesquiterpenoids (10%) were mainly derived from Formosan soft corals.

### 2.1. Lemnalia africana

**Figure 5:**
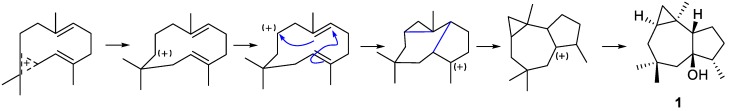
Plausible biogenetic pathway of compound **1**.

To the best of our knowledge, the first chemical investigation of *Lemnalia* (*L*. *africana*; collected around the island of Leti, province of Maluku, Indonesia) was conducted by Losman’s lab in 1974 [7]. Two years later, Karlsson reported the absolute configuration (AC) of the new type of tricyclic sesquiterpene, africanol (**1**) by single-crystal X-ray diffraction (XRD) analysis [8], determined to be 1*R*,5*S*,6*R*,7*S*,10*S* which was the enantiomer of the previously assigned structure. The biogenetic pathway of **1** was also proposed, as shown in Figure 5.

Although the first metabolite from Lemnalia was reported as a tricyclic sesquiterpenes (africanol), the most typical metabolites were sesquiterpenes with nardosinane skeleton (Scheme 1). In a study of Australian soft coral reported in 1980, Bowden and co-workers described the isolation and structural elucidation of two new nardosinane-type sesquiterpenes (**2** and **3**), along with a novel norsesquiterpene (**5**) from *L. africana* [9]. Besides, this study also highlighted that the skeleton of nardosinane-type sesquiterpenoids were of specific stereochemistry at C-4 and C-5 positions with *β*-orientation. In fact, this hypothesis was further evidenced by natural product studies of many other marine soft corals, such as *Paralemnalia thyrsoides* and *Nephthea chabroli* [10,11,12]. The two different collections of *L. africana*, namely G12607 and G12604, were collected in deep water by scuba or snorkeling over shallow reefs. The chemical structures and ACs of these compounds were further confirmed by XRD or/and chemical transformations. The second specimen of *L. africana* (G12604) afforded a predominant product named (2*R*,11*S*,12*R*)-lemnal-1(10)-ene-2,12-diol (**3**). In fact, the AC of hydroxyl groups at C-2 of **2** and **3** were assigned as 2*β* by the comparison of ^1^H NMR spectra between two isolates and a known compound from *L. carnosa* with a 2*α*-hydroxy group named lemnacarnol (**4**) [13], which was structurally characterized by XRD analysis. Finally, the most unexpected metabolite was a nornardosinane-type sesquiterpenoid named (2*R*, 7*S*)-7-formyloxy-2-hydroxy-12-nornardosin-1(10)-en-11-one (**5**) [9]. 

One year later in 1981, Izac and co-workers reported the discovery of two new sesquiterpenoids (**6** and **7**) with unprecedented ring systems and an eremophilane-derived sesquiterpene diol (**8**) from pacific-derived soft coral *L. africana* [14]. The animals were collected in Palau, Western Caroline Islands and a CHCl_3_/MeOH extract was chemically investigated. This research indicated the extension of the biosynthetic ability of soft coral *Lemnalia* by representing an additional skeleton (neolemnane-type) aside from the classic nardosinane-type sesquiterpenoid and africanol derivatives. The ring-enlarged neolemnane-type sesquiterpenoid also showed spectroscopic features similar to the nardosinane-type sesquiterpenoids with three methyl groups, and one tri-substituted carbon-carbon double bond. Based upon the biogenetic relationship of compounds with the nardosinane (as known as lemnalane) [15] sesquiterpenoids which are major components of *Lemnalia* species, it was suggested that the numbering sequence of ring-enlarged 6/8 ring system be changed, as shown in Scheme 1. Also, since the biogenetic relationship of the two different sesquiterpenoid types (nardosinane (lemnalane) and the new skeleton) are closely related, the new skeleton was named as neolemnane. The structural identification of compound **6** ((1*β*,2*Z*,4*β*,10*α*,12*β*)-4-acetoxy-10-hydroxy-neolemna-2,8-dien-5-one) was straightforward. Since it was obtained as a crystal and consequent XRD analysis allowed not only to unambiguous recognize its structure, but also to define its relative configuration as 1S*,4S*,10S*,12S*. From the less polar fraction, the diacetate **7** ((1*β*,2*Z*,4*β*,10*α*,12*β*)-4,10-diacetoxy-neolemna-2,8-dien-5-one) was also determined by NMR analysis. At last, the chemical structure of sesquiterpene diol was characterized as eremophila-6,10-diene-11,12-diol (**8**).

**Figure 6:**
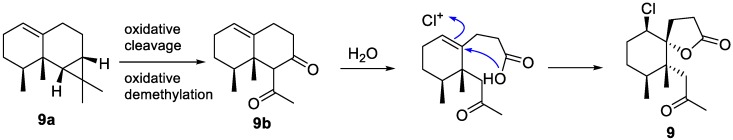
Plausible biogenetic pathway of **9**.

In 1992, a new halogenated norsesquiterpenoid, napalilactone (**9**), was isolated from the soft coral *L. africana* collected in Pohnpei, Federated States of Micronesia [16]. Despite the fact that the chlorine-containing metabolites are ubiquitous in marine-derived invertebrates, no chlorinated sesquiterpenoid or norsesquiterpenoid was reported from soft coral until napalilactone was reported. Along with the novel metabolite **9**, two known compounds (**10**–**11**) [17,18] with higher polarity were also isolated and identified. Biogenetically, the biosynthetic pathway of **9**, involving the intermediate (**9b**), a known norsesquiterpenoid that was generated from 1(10)-aristolene (**9a**) [15], is shown in Figure 6. The generation of **9** involved the hydrolysis of the *β*-diketone of **9b** followed by a haloperoxidase-generated chloronium ion-assisted lactonization. Several years later in 2002, Vyvyan and co-workers synthesized the spirolactone core of **9** in five steps from 2-methyl-2-cyclohexen-1-one or 2,3-dimethyl-2-cyclohexen-1-one [19].

In fact, the first biological investigation of terpenoids from *L. africana* was reported in 1993 by Scheuer’s lab [20]. In this study, the biological activity of an EtOAc extract of *L. africana* (collected from Pohnpei, Mincronesia) was tested. The extract with anti-leukemia activity in the P-388 assay afforded a total of sixteen sesquiterpenoids and norsesquiterpenoids, including six new compounds (**12**–**17**). These new compounds were identified as 4-acetoxy-2,8-neolemnadien-5-one (**12**), 12-acetoxy-1(10)-aristolene (**13**), 2,7-nardosinoxanedione (**14**), 4-acetoxy-6,10-guaiadiene (**15**), 1(10)-epoxy-2-hydroxy-12-nornardosin-7,11-dione (**16**, 6*β*; **17** 6*α*), respectively. The known compounds were readily identified as bicyclogermacrene (**18**) [18], germacrene D (**19**) [21], 1,6-germacradien-5-ol (**20**) [22], 1(11),5(12),6-germacratrien-2-ol acetate (**21**) [23], (1*β*,2*Z*,4*β*,10*α*,12*β*)-4-acetoxy-10-hydroxy-neolemna-2,8-dien-5-one (**6**) [14], lemnacarnol (**4**) [13], 2-oxolemnacarnol (**22**) [18,24], eremophila-6,10-diene-11,12-diol (**8**) [14], (1*β*,2*Z*,4*β*,10*α*,12*β*)-4,10-diacetoxy-neolemna-2,8-dien-5-one (**7**) [14], and 2-acetoxy-l(11),6-germacradien-5-ol (**23**) [22]. The main active compound in the extract was then found to be the known compound **8**. Compound **8** displayed cytotoxic activity against leukemia in the P-388 assay at 1 μg/mL whereas it had cytotoxicity in the CV-1 assay at 10 μg/mL. It is worth mentioning that the authors accomplished the isolation of terpenoids resulting in seven different skeletons, which further supported the chemical diversity and complexity of the secondary metabolites from marine soft coral *L. africana*.

The most recent chemical investigation of *L. africana* was in 2008 by Kashman’s lab. They reported the chemical structures of three new nornardosinanes named nardosinanols G–I (**24**–**26**), and one new tricyclic sesquiterpenoid named lemnafricanol (**27**) from a CH_2_Cl_2_ extract of *L. africana* [25]. It was described that the soft coral was collected at Fundu (deep gap south), Pemba Island, Tanzania. It was also noted that compounds **24** and **27** were found to be toxic to brine shrimp with LC_50_ values of 0.35 and 0.32 μM, respectively.

### 2.2. Lemnalia flava

During the studies of chemical composition in the soft coral *L. flava* collected on Shelly Reef, off Mombasa, Kenya, Kashman’s lab discovered four new diterpene glycosides (**28**–**31**) [26]. This research was the first chemical investigation of *L. flava*. During the investigation, the major composition of an EtOAc extract was determined to be lemnaflavoside (**28**). Unlike the abundance of lemnaflavoside from the sample, the other three metabolites were minor analogs with one hydroxyl group acetylated at various positions in the d-xylose moiety of **28**.

It was reported that a new ylangene-type sesquiterpenoid (**32**) was isolated from the Formosan soft coral *L. flava* [27] along with two related known ones, lemnalol (**33**) [28] and cervicol (**34**) [29]. It is worth mentioning that the parent hydrocarbon of ylangane is a stereoisomer of *α*-copaane as regards the orientation of the isopropyl group at C-8 (*α*-orientation for ylangane, *β*-orientation for copaane) [30]. The animals were collected at the Green Island located in the southeast coast of Taiwan by scuba. The resulting new metabolite (**32**) was identified as (1*S*,2*S*,4*R*,6*S*,7*R*,8*S*)-4*α*-formyloxy-*β*-ylangene by NMR analysis and chemical transformation. Biologically, the authors evaluated the in vitro anti-inflammatory activity of these metabolites. The new compound **32** was found to be able to significantly inhibit iNOS (inducible nitric oxide synthetase) protein expression and **34** was shown to be active in inhibiting both iNOS and COX-2 (cyclooxygenase-2) proteins expression.

**Figure 7:**
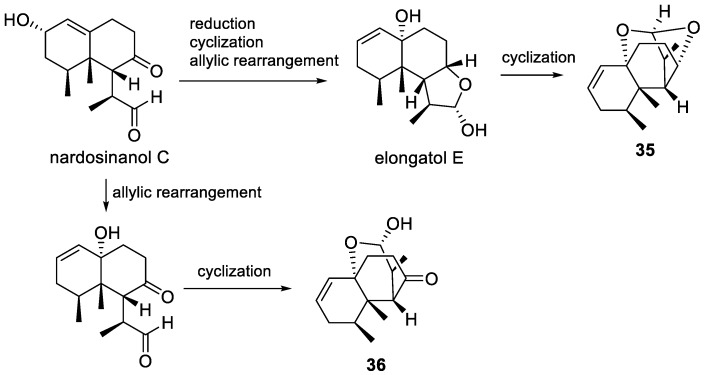
Plausible biogenetic pathway of **35** and **36**.

Another discovery of sesquiterpenoid from Formosan soft coral *L. flava* was reported by Sheu’s lab in 2011. Still, the animals were collected by hand using scuba off the coast of Green Island, Taiwan. Four new nardosinane-type sesquiterpenoids, flavalins A–D (**35**–**38**) were isolated [31]. The structures were elucidated by extensive spectroscopic analyses. Flavalin B (**36**) was further determined by XRD analysis. The oxidative patterns of these polyoxygenated nardosinane-type sesquiterpenoids, especially compound **35**, which featured a unique tetracyclic oxacage ring, was different from those secondary metabolites discovered from soft coral of the genus *Lemnalia* before. From biogenetical standpoint, the more complex products could be generated from normal nardosinane-type sesquiterpenoid, such as nardosinanol C (Figure 7) [25], a proposed precursor of these compounds, by allylic rearrangement and cyclization. These metabolites were shown to possess different biological activities, for instance, flavalin A (**35**) was found to exhibit inhibition of lipopolysaccharide (LPS)-induced iNOS and COX-2 protein expression in a dose-dependent manner with ED_50_ values of 4.8 ± 0.3 μg/mL and 6.2 ± 0.6 μg/mL, respectively. Besides, both flavalins A and B (**35** and **36**) showed significant neuroprotective activity. Pretreatment of **35** and **36** at different concentrations on SH-SY5Y cells significantly reduced 6-hydroxydopamine (6-OHDA) induced cytotoxicity at a concentration of 20 μM.

In the same year, Sheu’s lab also reported the continuing study of the sample collected at Green Island. A total of four new nardosinanes named flavalins E–H (**39**–**42**) [32] and two new nornardosinanes named flavalins I (**43**) and J (**44**), along with five known compounds, 2-oxolemnacarnol (**22**), lemnacarnol (**4**), armatin F (**45**) [29], (2*R*)-2-hydroxylemnal-1(10)-en-12-one (**46**) [24], and laevinol B (**47**) [33] were obtained. For the first time, the AC of compound **46** was assigned as *R* configuration at C-2 by Mosher’s method. The presence of both the C-12 methoxy epimers (**41**, **45**) and the use of MeOH during the purification suggest that methoxy-containing compounds **39**, **41**, **42**, and **45** might be artifacts. A further experiment was conducted to address this issue. It was reported that the methoxy group in flavalin E (**39**) was not obtained from 2-oxolemnacarnol (**22**) with the presence of MeOH in silica gel for ten days. Thus, it was believed that the methoxy containing compounds were genuine natural products and the methoxy group was obtained during the generation of secondary metabolites in natural environment. All of the molecules were evaluated for cytotoxicity against a panel of cancer cell lines, including human breast carcinoma (MCF-7), human colon carcinoma (WiDr), human laryngeal carcinoma (HEp 2), human medulloblastoma (Daoy), T-cell acute lymphoblastic leukemia (CCRF-CEM), colon adenocarcinoma (DLD-1), human promyelocytic leukemia (HL-60), and murine leukemia (P388D1) but none displayed cytotoxicity at 20 μg/mL.

### 2.3. Lemnalia philippinensis

Two oxygenated ylangene sesquiterpenoids, philippinlins A (**48**) and B (**49**) [34], and one known compound, lemnalol (**33**) [28], were obtained from the soft coral *L. philippinensis*. The animals were collected from the coast of Lanyu, Taiwan. It was noted that the ACs of two new products were tentatively assigned as same as lemnalol (**33**), based mainly on the NMR analysis and biogenetic consideration. As part of their biological experiment, the cytotoxicity of philippinlins and lemnalol against human liver carcinoma (HepG2), human breast carcinoma (MDA-MB231) and human lung adenocarcinoma epithelial cells (A549) were evaluated. Philippinlin A was shown to be cytotoxic against HepG2, MDA-MB231, and A549 cancer cell lines with IC_50_ values of 16.0, 16.3, and 15.8 μg/mL, respectively.

**Figure 8:**
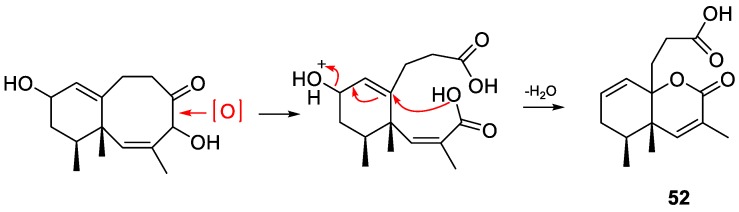
Plausible biogenetic pathway of **52**.

The continuing chemical investigation of the Formosan soft coral *L. philippinensis*, with the same collection mentioned above, gave three new sesquiterpenoids, including two new eremophilane-type metabolites, philippinlins C (**50**) and D (**51**), and a new 4,5-seconeolemnane, philippinlin E (**52**) [35]. Besides, one known eremophilane-type compound, eremophila-6,10-diene-11,12-diol (**8**), and one known neolemnane-type compound, (1*β*,2*Z*,4*β*,10*α*,12*β*)-4-acetoxy-10-hydroxy-neolemna-2,8-dien-5-one (**6**), were also identified from the same extract. Although the NMR spectroscopic analyses of the two new eremophilane-type compounds were able to establish the gross structures, the relative configuration of the chiral center at C-11 remained unknown. In this paper, the authors also focus on the plausible biosynthetic pathway of philippinlin E (**52**). In fact, it was proposed that **52** was generated from the oxidation with ring cleavage of a neolemnane, followed by the subsequent nucleophilic conjugate substitution (Figure 8). As a routine screening of biological activity, all the compounds were tested against HepG2, MDA-MB231, and A549 carcinoma cells yet none of them were cytotoxic.

### 2.4. Lemnalia cervicornis

The search for natural products from Australian soft coral *L. cervicornis* afforded thirteen novel sesquiterpenoids whose structures were based on the cadinane. The soft corals were collected at Broadhurst Reef, 60 km E.N.E. of Townsville. Eleven aromatic metabolites (**53**–**63**) and two minor metabolites with more saturated structures (**64**–**65**) were isolated [36]. They were identified as (1*S*,4*R*)-7-hydroxycalamenen-3-one (**53**), (1*S*,4*R*)-7-acetoxycalamenen-3-one (**54**), (1*S*,4*R*)-7-methoxycalamenen-3-one (**55**), (1*S*,3*R*,4*S*)-calamenen-3,7-diol (**56**), (1*S*)-7-methoxy-1,2-dihydrocadalene (**57**), (1*S*,4*S*)-7-methoxycalamenen-4-ol (**58**), (1*S*,4*R*)-7-methoxycalamenen-4-ol (**59**), (1*S*,3*R*,4*R*)-calamenen-3,4,7-triol (**60**), (1*S*,3*R*,4*S*)-calamenen-3,4,7-triol (**61**), (1*S*,3*R*,4*R*)-methoxycalamenen-3-ol (**62**), (1*S*,3*R*,4*R*)-7-methoxycalamenen-3-yl acetate (**63**), 12-hydroxyzonarene (**64**), and 12-acetoxyzonarene (**65**). The chemical transformations of these compounds were also carried out. 

Four ylangene-type sesquiterpenoids, including two new compounds cervicol (**34**) and isolemnalol (**66**) [29], two known lemnalol (**33**) [28] and 4-oxo-*α*-ylangene (**67**) [37] were isolated from Formosan soft coral *L. cervicornis*. Animals were collected at Green Island, off Taiwan. The in vitro cytotoxicity against selected cancer cells was measured. Only the known compound lemnalol exhibited cytotoxicity against P-388 and HT-29 with ED_50_ values of 16.3 μM and 10.5 μM, respectively. In 2008, Wen and co-workers tested the anti-inflammatory and analgesic effects of a natural marine compound lemnalol (**33**) [28] isolated from Formosan soft coral *L. cervicornis* [38]. The anti-inflammatory and anti-nociceptive properties were investigated in LPS-stimulated RAW 264.7 cells and carrageenan-injected rats. The results indicated the lemnalol could be a potent therapeutic agent for inflammatory pain.

### 2.5. Lemnalia bournei

Lemnabourside (**68**), a novel diterpene glycoside with d-glucose attached to a diterpene aldehyde through an acetal linkage, was isolated from the soft coral of *L. bournei* [39]. The fresh Chinese soft corals were collected from South China Sea near the Xisha Islands. The stereochemistry of **68** was solved by careful interpretation of the well-resolved NOESY correlations and its ^1^H-^1^H coupling constants. Finally, only the ACs at C-15 and C-16 could be determined as 15*R*, 16*S*, while the other chiral centers at C-5, C-6, C-10, C-11 remained unknown. Four years later in 1998, the Zhang lab further reported two new diterpene glycosides, lemnaboursides B (**69**) and C (**70**), from South China Sea soft coral *L. bournei* [40]. In this article, the animals were collected from the same location as a previous collection aforementioned. Structurally, the NMR spectroscopic data of these two compounds showed high similarity with the known compound **68**. Detailed analysis of these data revealed the two new compounds were analogs of **68** with monoacetylation of the sugar residue at different hydroxyl groups. Biologically, three metabolites only showed weak cytotoxicity against HepA (hepatoma ascites), S_180_A (sarcoma 180 ascites), and EAC (Ehrlich ascites carcinoma) cells with IC_50_ values ranging from 27.4 to 186.9 μg/mL.

### 2.6. Lemnalia tenuis

The first discovery and structure elucidation of the well-known metabolite lemnalol (**33**) was from the soft coral *L. tenuis* Verseveldt, reported by Kikuchi and co-workers [28]. The animals were collected from the coral reefs of Ishigaki Island (Okinawa, Japan) and then extracted by EtOAc. The chemical structure of **33** was solved by a combination of NMR analysis, chemical transformation, and XRD analysis. A year later in 1983, Kikuchi and co-workers tested the cytotoxic activities of lemnalol and its derivatives [41]. The new compound lemnalol could activate peritoneal exudate cells (PEC), exhibiting cytotoxic effects in vitro. Surprisingly, compound **71**, the C-4 epimer of lemnalol showed no PEC-mediated or direct cytotoxicity while the derivative with an *α*,*β*-unsaturated ketone (**72**) showed direct cytotoxicity in vitro. Based on the biological results, it was reasonable to speculate that the 4*α*-configuration is quite essential for in vitro cytotoxic activity by PEC-mediated cytotoxicity.

### 2.7. Lemnalia laevis

The only chemical study of soft coral *L. laevis* was reported in 2005 by Duh and co-workers. They investigated the chemical composition of the animals collected at Green Island, off Taiwan, which resulted eight new nornardosinane sesquiterpenoids, laevinols A (**73**), B (**47**), C–H (**74**–**79**), a new neolemnane sesquiterpenoid, laevinone A (**80**) [33], along with two known compounds, eremophila-6,10-diene-11,12-diol (**8**) [14] and 6*β*-acetyl-4*β*,5*β*-dimethyl-1(10)*α*-epoxy-2*β*-hydroxy-7-oxodecalin (**81**) [20]. Among all the metabolites, only the known compound **8** exhibited in vitro cytotoxicity against P-388 (mouse lymphocytic leukemia) cells and HT-29 (human colon adenocarcinoma) cells with ED_50_ values of 0.21 and 0.33 μg/mL, respectively.

### 2.8. Lemnalia humesi

The Australian soft corals *L. humesi* gave two new aristolane-type sesquiterpenoids, (4*R*,5*S*,6*R*,7*S*)-aristol-9-en-3-one (**82**) and (3*S*,4*R*,5*S*,6*R*,7*S*)-aristol-9-en-3-ol (**83**) [42]. The new metabolites were identified by NMR spectroscopic data and further confirmed by chemical conversion. Besides, the AC of the hydroxyl group at C-3 in **83** was determined as 3*S*.

### 2.9. Other Lemnalia sp.

Lemnalosides A–D (**84**–**87**), four bisabolane-type bicyclic diterpene glycosides and one known diterpene lemnabourside (**69**), were isolated from marine soft coral *Lemnalia* collected from South China Sea, Malaysia [43]. The CH_2_Cl_2_-MeOH extract exhibited activity in hyphae formation inhibition (HFI) assay against *Streptomyces* 85E with a concentration of 20 μg/disk. Due to the limited material obtained from the purification, only **84**–**86** and **69** were evaluated for HFI activity. Lemnaloside B (**85**) showed the best activity with an inhibitory clear zone with 18 mm at a concentration of 20 μg/disk. Besides, the clear inhibitory zone was still visible at the concentration of 2.5 μg/disk. Lemnaloside A (**84**) only showed moderate activity (11 mm) with a concentration of 2.5 μg/disk while no activity was observed for compounds **86** and **69** at the same concentration. Extraction of *Lemnalia* sp. and isolation of a new nardosinane-type sesquiterpenoid, nardosinanol A (**88**), was also described in Kashman’s paper in 2008 [25]. The soft coral was obtained from Kitagamwa, southern Kenya. The chemical structure of nardosinanol A was tentatively assigned as 2-deoxy-12-oxolemnacarnol.

## 3. Conclusions and Perspectives

Extensive efforts have been devoted to implementing chemical investigations of *Lemnalia* during the past 44 years from 1974 to present. One of the most significant findings to emerge from these studies is the chemical diversity and biological activities of the secondary metabolites from marine soft coral *Lemnalia*. This paper presents an exhaustive review of these studies including the discovery of small molecules and their biological evaluation.

It is worth noting that the plausible biogenetic pathways of several sesquiterpenoids were proposed. Thus, it could inspire biomimetic syntheses and compound interconversion to scale up the available amounts for further specific pharmacological and toxicological evaluations. Another outlook worth mentioning is the possible biogenetic relationship between some metabolites in this genus. To the best of our knowledge, the production of these diverse metabolites may be due to the unique adaptive strategy of soft corals living in various marine environments [44,45,46]. For instance, diterpene glycosides were mainly discovered in South China Sea, whereas nornardosinane sesquiterpenoids were mostly isolated from Formosan soft corals. In the future, more chemical investigations of soft corals from specific marine ecological environment are warranted for the demonstration of the chemical ecological significance of these metabolites in the life cycle of *Lemnalia*. Furthermore, special attention should be paid to symbiotic microorganisms of *Lemnalia* owing to the fact that some therapeutic agents of marine invertebrates are biosynthesized by their symbiotic microbes. We hold the belief that symbiotic microbes in *Lemnalia* have taken part in the generation of some metabolites, which could be another direction for the future study. All these efforts would speed up the discovery of functional metabolites, not only from *Lemnalia*, but also from all precious marine resources.

## Figures and Tables

**Table 1:** *Lemnalia*-derived secondary metabolites.

Organism	Locality	Secondary Metabolite	Reference
*Lemnalia africana*	Maluku, Indonesia	africanol (**1**)	[7,8]
	Australia	(2*R*,7*S*,11*R*)-7-acetoxy-2-hydroxynardosin-1(10)-en-12-al (**2**), (2*R*,11*S*,12*R*)-lemnal-1(10)-ene-2,12-diol (**3**), (2*R*,7*S*)-7-formyloxy-2-hydroxy-12-nornardosin-1(10)-en-11-one (**5**)	[9]
	Palau, Western Caroline Islands	(1*β*,2*Z*,4*β*,10*α*,12*β*)-4-acetoxy-10-hydroxy-neolemna-2,8-dien-5-one (**6**), (1*β*,2*Z*,4*β*,10*α*,12*β*)-4,10-diacetoxy-neolemna-2,8-dien-5-one (**7**), eremophila-6,10-diene-11,12-diol (**8**)	[14]
	Pohnpei, Micronesia	napalilactone (**9**), compounds **10**–**11**	[16]
	Pohnpei, Mincronesia	4-acetoxy-2,8-neolemnadien-5-one (**12**), 12-acetoxy-1(10)-aristolene (**13**), 2,7-nardosinoxanedione (**14**), 4-acetoxy-6,10-guaiadiene (**15**), 1(10)-epoxy-2-hydroxy-12-nornardosin-7,11-dione (**16**, 6*β*; **17** 6*α*), bicyclogermacrene (**18**), germacrene D (**19**), 1,6-germacradien-5-ol (**20**), 1(11),5(12),6-germacratrien-2-ol acetate (**21**), 4-acetoxy-l0-hydroxy-5-oxo-2,8-neolemnadiene (**6**), lemnacarnol (**4**), 2-oxolemnacarnol (**22**), 11,12-dihydroxy-6,10-eremophiladiene (**8**), 4,10-diacetoxy-5-oxo-2,8-neolemnadiene (**7**), 2-acetoxy-l(11),6-germacradien-5-ol (**23**)	[20]
	Fundu, Tanzania	nardosinanols G–I (**24**–**26**), lemnafricanol (**27**)	[25]
*L. flava*	Mombasa, Kenya	lemnaflavoside (**28**), compounds **29**–**31**	[26]
	Green Island, Taiwan	(1*S*,2*S*,4*R*,6*S*,7*R*,8*S*)-4*α*-formyloxy-*β*-ylangene (**32**), lemnalol (**33**), cervicol (**34**)	[27]
	Green Island, Taiwan	flavalins A–D (**35**–**38**)	[31]
	Green Island, Taiwan	flavalins E−H (**39**–**42**), flavalins I (**43**) and J (**44**), 2-oxolemnacarnol (**22**), lemnacarnol (**4**), armatin F (**45**), (2*R*)-2-hydroxylemnal-1(10)-en-12-one (**46**), and laevinol B (**47**)	[32]
*L*. *philippinensis*	Lanyu, Taiwan	philippinlins A (**48**) and B (**49**), and lemnalol (**33**)	[34]
	Lanyu, Taiwan	philippinlins C (**50**) and D (**51**), philippinlin E (**52**), 11,12-dihydroxy-6,10-eremophiladiene (**8**), 4-acetoxy-10-hydroxy-5-oxo-2,8-neolemnadiene (**6**)	[35]
*L. cervicornis*	Broadhurst Reef, Townsville	(1*S*,4*R*)-7-hydroxycalamenen-3-one (**53**), (1*S*,4*R*)-7-acetoxycalamenen-3-one (**54**), (1*S*,4*R*)-7-methoxycalamenen-3-one (**55**), (1*S*,3*R*,4*S*)-calamenen-3,7-diol (**56**), (1*S*)-7-methoxy-1,2-dihydrocadalene (**57**), (1*S*,4*S*)-7-methoxycalamenen-4-ol (**58**), (1*S*,4*R*)-7-methoxycalamenen-4-ol (**59**), (1*S*,3*R*,4*R*)-calamenen-3,4,7-triol (**60**), (1*S*,3*R*,4*S*)-calamenen-3,4,7-triol (**61**), (1*S*,3*R*,4*R*)-methoxycalamenen-3-ol (**62**), (1*S*,3*R*,4*R*)-7-methoxycalamenen-3-yl acetate (**63**), 12-hydroxyzonarene (**64**), and 12-acetoxyzonarene (**65**)	[36]
	Green Island, Taiwan	cervicol (**34**), isolemnalol (**66**), lemnalol (**33**), 4-oxo-*α*-ylangene (**67**)	[37]
*L. bournei*	South China Sea	lemnabourside (**68**)	[39]
	South China Sea	lemnaboursides B (**69**) and C (**70**)	[40]
*L. tenuis*	Okinawa, Japan	lemnalol (**33**), compounds **71** and **72**	[28]
*L. laevis*	Green Island, off Taiwan	laevinols A (**73**), B (**47**), C–H (**74**–**79**), laevinone A (**80**), 11,12-dihydroxy-6,10-eremophiladiene (**8**), and 6*β*-acetyl-4*β*,5*β*-dimethyl-1(10)*α*-epoxy-2*β*-hydroxy-7-oxodecalin (**81**)	[33]
*L. humesi*	Australia	(4*R*,5*S*,6*R*,7*S*)-aristol-9-en-3-one (**82**) and (3*S*,4*R*,5*S*,6*R*,7*S*)-aristol-9-en-3-ol (**83**)	[42]
Unclassified *Lemnalia* sp.	South China Sea	lemnalosides A–D (**84**–**87**), lemnabourside (**68**)	[43]
	Fundu, Tanzania	nardosinanol A (**88**)	[25]
